# In Vitro Anti-Inflammatory Activity of* Morus alba* L. Stem Extract in LPS-Stimulated RAW 264.7 Cells

**DOI:** 10.1155/2017/3928956

**Published:** 2017-06-08

**Authors:** Nattaporn Soonthornsit, Chetsadaporn Pitaksutheepong, Warinkarn Hemstapat, Pongsak Utaisincharoen, Tasana Pitaksuteepong

**Affiliations:** ^1^Department of Pharmaceutical Technology, Faculty of Pharmaceutical Sciences and Center of Excellence for Innovation in Chemistry, Naresuan University, Tha Pho, Mueang Phitsanulok, Phitsanulok 65000, Thailand; ^2^Food Biotechnology Research Unit, National Center for Genetic Engineering and Biotechnology (BIOTEC), 113 Thailand Science Park, Phahonyothin Road, Khlong Nueng, Khlong Luang, Pathum Thani 12120, Thailand; ^3^Department of Pharmacology, Faculty of Science, Mahidol University, Rama VI Road, Ratchathewi, Bangkok 10400, Thailand; ^4^Department of Microbiology, Faculty of Science, Mahidol University, Rama VI Road, Ratchathewi, Bangkok 10400, Thailand

## Abstract

*Morus alba* L., also known as white mulberry or Mhon, has long been used in traditional medicines. This study was aimed to investigate anti-inflammatory activities of mulberry stem ethanolic extract (MSE) in lipopolysaccharide- (LPS-) stimulated RAW 264.7 macrophage cell line. The MSE was first prepared and then investigated for cell viability using the MTT assay. The anti-inflammatory activities were investigated through the inhibition of inducible nitric oxide synthase (iNOS), cyclooxygenase- (COX-) 2 mRNA expression, and iNOS protein expression using reverse transcription-polymerase chain reaction (RT-PCR) assay and immunoblotting analysis, respectively. The inhibition of nitric oxide production of the MSE was also investigated using the Griess reaction assay. The MSE concentration ranging from 10 to 40 *µ*g/ml yielded cell viability higher than 80%. The MSE at concentrations of 20 and 40 *µ*g/ml demonstrated anti-inflammatory activity through the inhibition of nitric oxide production via suppression of both the iNOS mRNA and protein. It was also found to inhibit the expression of COX-2 mRNA in LPS-induced RAW 264.7 cells. This study is the first to report the anti-inflammatory potential of the extract prepared from the stem of mulberry.

## 1. Introduction

*Morus alba* (Moraceae) is known as mulberry or Mhon in Thai. Its leaves, fruit, and bark have long been used in traditional Chinese medicine to treat fever, improve eyesight, strengthen joints, and lower blood pressure [1]. The leaves are the most widely used and are consumed as an antihyperglycemic supplement in Korea and Japan [1]. Cultivation of mulberry trees in Thailand is also widespread, particularly for the leaves to feed silkworms. The twigs and stems are often pruned, cut as normal cultivation practice, and used for firewood but are rarely used for other purposes, such as medicinal use.

A natural chemical compound found in the twigs and stems of mulberry trees is oxyresveratrol or* trans*-2, 3′, 4, 5′-tetrahydroxystilbene. This compound has been reported to possess antioxidative and radical scavenging activities with IC_50_ value of 3.6 ± 0.0 *μ*M and 15.1 ± 2.3 *μ*M using FeSO_4_/H_2_O_2_-induced lipid peroxidation in rat liver microsomes and DPPH assay, respectively [2]. It has also been reported to have anti-inflammatory activity by inhibiting the production of nitrite, prostaglandin E_2_, and inducible nitric oxide synthase (iNOS) expression in the LPS-activated RAW 264.7 macrophage cells through the NF-*κ*B activation pathway [2]. In addition, oxyresveratrol has been shown to inhibit the rat paw edema induced by carrageenan in a dose-dependent manner [2]. Apart from the NF-*κ*B signaling pathway, the anti-inflammatory effect of oxyresveratrol in LPS-induced RAW264.7 cells has recently been reported as also occurring through the inhibition of the mitogen-activated protein kinase (MAPK) pathway [3].

Our previous study [4] found different amounts of oxyresveratrol in various parts of the mulberry tree, and the ethanolic extract obtained from the mulberry stems has the highest amount of oxyresveratrol compared to that obtained from the twigs, with the least amount in the leaves. Therefore, we hypothesized that the stem wood of mulberry trees, which was left unused, would also have anti-inflammatory activity.

Inflammation is the body's protective response that intended to eliminate the initial cause of cell injury. However, sometimes, it can also become self-perpetuating and cause further inflammation. Chronic inflammation is involved in many diseases, for example, ulcerative colitis, Alzheimer's, dermatitis, cardiovascular diseases, cancer, inflammatory bowel disease (IBD), systemic lupus erythematous (SLE), osteoarthritis (OA), and rheumatoid arthritis (RA) [5]. Once macrophages are elicited, inflammatory mediators including nitric oxide and proinflammatory mediators such as iNOS and COX-2 are produced. There are few reports [1, 6] on the anti-inflammatory effect of the stems of mulberry trees. Therefore, this study was aimed to investigate the anti-inflammatory activities of mulberry stem extract (the MSE) through the inhibition of iNOS and COX-2 expression and NO production in LPS-stimulated RAW 264.7 macrophage cells.

## 2. Materials and Methods

### 2.1. Materials

Oxyresveratrol, isolated and purified from* Artocarpus lakoocha* heartwood or Puag-Haad, was prepared in-house using the method as previously described [7]. HPLC grade acetonitrile was purchased from RCI Labscan (Bangkok, Thailand). A mouse macrophage cell line (RAW 264.7) was obtained from the American Type Culture Collection (ATCC #TIB-71, Manassas, Virginia, USA). Lipopolysaccharide (LPS), 3-(4, 5-dimethylthiazol-2-yl)-2, 5-diphenyltetrazolium bromide (MTT), and dimethylsulfoxide (DMSO) were purchased from Sigma-Aldrich (Saint Louis, Missouri, USA). RNAspin Mini RNA Isolation Kit was purchased from GE Healthcare (Buckinghamshire, UK). iScript™ cDNA Synthesis Kit was purchased from Bio-Rad Laboratories (Hercules, California, USA). dNTPs were purchased from Fermentas (Vilnius, Lithuania). Taq DNA polymerase was purchased from Invitrogen (California, USA). Griess Reagent Kit was purchased from Molecular Probes, Inc. (Eugene, Oregon, USA). Dulbecco's modified Eagle's medium (DMEM) and fetal bovine serum (FBS) were purchased from HyClone (Logan, Utah, USA). L-Glutamine was purchased from Biological Industries (Kibbutz Beit Haemek, Israel). Blocking solution was purchased from Roche Diagnostics (Mannheim, Germany). Specific monoclonal rabbit antibody to mouse iNOS was purchased from Santa Cruz Biotechnology (Santa Cruz, California, USA). Specific monoclonal mouse antibody to *β*-actin was purchased from R&D systems (Minnesota, USA). Horseradish peroxidase- (HRP-) conjugated goat anti-rabbit IgG and HRP-conjugated rabbit anti-mouse IgG were purchased from Pierce (Rockford, Illinois, USA).

### 2.2. Preparation of the MSE

Fresh mulberry stems (var. Buriram 60) were supplied by the Queen Sirikit Sericulture Center, Tak Province, Thailand. A voucher specimen was verified by Dr. Pranee Nangngam and deposited at the Faculty of Science, Naresuan University (voucher specimen number 004067). The chopped and dried inner wood of the mulberry stems (3,700 g) was macerated in 4 L of 80% ethanol. After filtration, the filtrate was evaporated under reduced pressure using a rotary evaporator (Buchi Rotavapor R-114, Flawil, Switzerland) and continued drying using a water bath (M25 Lauda, Deutschland, Germany). The percentage yield of the extract was calculated using the following equation:

(1) % yield=Dried  weight  of  crude  extractDried  weight  of  chopped-dried  plant×100.

### 2.3. High Performance Liquid Chromatography (HPLC) Analysis of Oxyresveratrol in the MSE

Oxyresveratrol was used as the marker compound for the quantitative HPLC analysis of MSE using the method described by Yhirayha [7]. Standard stock solution 1 mg/ml of oxyresveratrol was prepared by dissolving in 80% ethanol. The stock solution was further diluted to 5 standard working solutions over the concentrations range of 0.0005 to 0.05 mg/ml using 80% ethanol. An HPLC system (LC-20AT, Shimadzu, Kyoto, Japan), equipped with a UV-Vis detector (SPD-20A, Shimadzu, Japan), an autosampler (SIL-10ADVP, Shimadzu, Kyoto, Japan), and a column oven, was used. Analysis was performed on a C18 boned-silica gel column (Gemini, 5 u, 150 × 4.6 mm, Phenomenex, Torrance, USA) in the isocratic mode. Acetonitrile mixed with 0.05 M phosphate buffer pH 3 (13 ratio) was used as the mobile phase at a flow rate of 1 ml/min. UV detector wavelength of 320 nm with column oven temperature of 30°C was set. The injection volume was 20 *µ*l. Run time was set at 13 min. The stock solution of the extract was prepared in 80% ethanol at concentration of 0.25 mg/ml.

### 2.4. Thin Layer Chromatography (TLC) Analysis

The stock solutions of the crude extract and oxyresveratrol were prepared by dissolving in ethanol at concentration 6 mg/ml and 1 mg/ml, respectively. Ascending TLC analysis was performed using silica gel 60 F_254_ (Merck, Darmstadt, Germany) as a stationary phase and methanol : chloroform (15 : 85% v/v) as a mobile phase. Small amount of samples was spotted at about 0.5 cm from the bottom of the TLC plate and then placed in the chamber which was left to saturate with solvent vapor for 15 min. The developing time was allowed for 5 min. After that, the plate was removed from the chamber and air-dried. The TLC spots were viewed under UV light at 254 and 366 nm. Then, anisaldehyde reagent was sprayed on TLC plate and then heated continuously to 100°C in order to visualize oxyresveratrol. The colored spots appeared were marked and the retention factor (*R*_*f*_) was calculated from the following equation:

(2) $$\begin{array}{c} R_{f}=\frac{distance\;\;traveled\;\;by\;\;a\;\;compound}{\text{distance traveled by solvent front}}. \end{array}$$

### 2.5. Anti-Inflammatory Activity of the MSE

#### 2.5.1. Sample Preparation

The dried crude extract was dissolved in 0.5% DMSO to make a final concentration of 1 mg/ml and then filtered through 0.22 *µ*m nylon membrane (VertiClean™, Vertical Chromatography Co., Ltd., Bangkok, Thailand) before further analysis. Oxyresveratrol, a biological marker in this study, was also prepared in the same manner as the extract.

#### 2.5.2. Cell Culture and Viability

The RAW 264.7 cells were cultured in DMEM supplemented with 10% FBS and 1% L-glutamine and incubated at 37°C in 5% CO_2_. To determine cell viability, RAW 264.7 cells were seeded in a 96-well plate at a density of 3 × 10^4^ cells/well. After overnight incubation, the cells were treated with various concentrations of samples in the presence of 100 ng/ml LPS and incubated for 24 h. Cell viability was determined by MTT assay.

#### 2.5.3. Reverse Transcription-Polymerase Chain Reaction (RT-PCR) Assay

RAW 264.7 cells were seeded in a 6-well plate at a concentration of 2.5 × 10^5^ cells/ml. After overnight incubation, the cells were resuspended in 1 ml of supplemented DMEM and pretreated with various concentrations of samples for 2 h. LPS 100 ng/ml was then added and further incubated for 6 h. The cells were then washed twice with 1 ml PBS, harvested in 1 ml PBS, and subjected to RT-PCR analysis. Total RNA from the stimulated cells was extracted using RNAspin Mini RNA Isolation Kit according to the manufacturer's instruction. cDNA was synthesized using iScript cDNA Synthesis Kit. The TGradient thermal cycler 96 (Biometra, Goettingen, Germany) was used for PCR amplification. The PCR mixture contained 50 ng cDNA, 1x PCR buffer, 1.25 mM MgCl_2_, 100 *μ*M dNTPs, 450 nM of each primer, and 1 U Taq polymerase. The primers and PCR conditions used for amplification of iNOS, COX-2, and *β*-actin genes are shown in Table 1. The amplified products were separated on 1.5% (w/v) agarose gel. After gel electrophoresis was completed, the gels were stained with ethidium bromide and the PCR products were visualized under an UV illuminator (GeneSys, Rochester, New York, USA). The band intensity of iNOS and COX-2 genes was then normalized to *β*-actin and calculated as the relative expression.

#### 2.5.4. Immunoblotting Analysis

RAW 264.7 cells were seeded in a 6-well plate at a concentration of 1.9 × 10^5^ cells/ml. After overnight incubation, they were resuspended in 1 ml of supplemented DMEM and pretreated with various concentrations of samples for 2 h before LPS (100 ng/ml) was added. After 16 h of incubation, the cell supernatants were collected and subjected to NO production analysis (see Section 2.5.5). The cells were washed twice with PBS and resuspended in 1 ml PBS. Following centrifugation of the cell sample at 10,000 rpm, the cell pellet was lysed in lysis buffer, pH 6.8. The mixtures were sonicated on ice for 1 min and then heated for 5 min in a dry bath incubator (Boekel Scientific, Pennsylvania, USA). The mixture was centrifuged at 10,000 rpm at 4°C for 5 min. The cell lysates were electrophoresed on 8% SDS-PAGE and then electrotransferred to a nitrocellulose membrane. The membrane was blocked with 5% blocking solution (Roche Diagnostics, Mannheim, Germany) in PBS for 1 h before incubating at 4°C overnight with specific monoclonal rabbit antibody to mouse iNOS and specific monoclonal mouse antibody to *β*-actin. The concentration of each antibody was used according to the manufacturer's recommendation. The membranes were washed 3 times with 0.1% Tween 20 in PBS for 15 min, followed by reacting with HRP-conjugated goat anti-rabbit IgG (for iNOS) and HRP-conjugated rabbit anti-mouse IgG (for *β*-actin) at room temperature for 1 h. After that, the membrane was washed 4 times (20 min each wash) with 0.1% Tween 20 in PBS. Protein bands were detected by enhanced chemiluminescence as recommended by the manufacturer (Roche Diagnostics, Mannheim, Germany) after exposure to hyperfilm (GE Healthcare, Buckinghamshire, UK). The iNOS immunoblot signals were compared with *β*-actin and calculated as the relative protein expression.

#### 2.5.5. NO Production Assay

As detailed above, the nitrite concentration in cell supernatant was measured as an indicator of NO production using the Griess Reagent Kit. Briefly, 150 *µ*l of each supernatant was mixed with 20 *µ*l of Griess reagent (1% sulfanilamide in 5% phosphoric and 0.1% naphthylethylenediamine dihydrochloride in water) and 130 *µ*l of distilled water in a 96-well plate. The mixtures were incubated at room temperature for 30 min and then the absorbance of each well was determined at 548 nm using a microplate reader. The amount of nitrite in samples was back-calculated from a sodium nitrite calibration curve (0–100 *µ*M).

### 2.6. Statistical Analysis

All data were expressed as mean ± standard deviation (SD). Results were obtained from at least three independent experiments, each performed in triplicate. Statistical analysis was determined using one-way analysis of variance (ANOVA) followed by the Tukey's test for multiple comparisons (GraphPad Prism 6.0, GraphPad Software Inc., San Diego, USA). *P* values less than or equal to 0.05 were considered significant.

## 3. Results and Discussion

The leaves, fruit, and bark of mulberry have long been used to treat various illnesses and to improve health functions. However, few reports have investigated the health benefits of the stem wood of mulberry which is abundantly available in Thailand. The anti-inflammatory effects were the biological activity particularly investigated in this study.

### 3.1. HPLC Analysis of Oxyresveratrol in the MSE

First, the MSE was prepared. Ethanolic extract was obtained as a powder, which had a dark brown color, with a percentage yield of 5.13% of the raw woody material. The amount of oxyresveratrol in the MSE was analyzed by HPLC. The calculated amount of oxyresveratrol in the MSE was 17.87 ± 0.61% [9].

### 3.2. TLC Analysis

**Figure 1:**
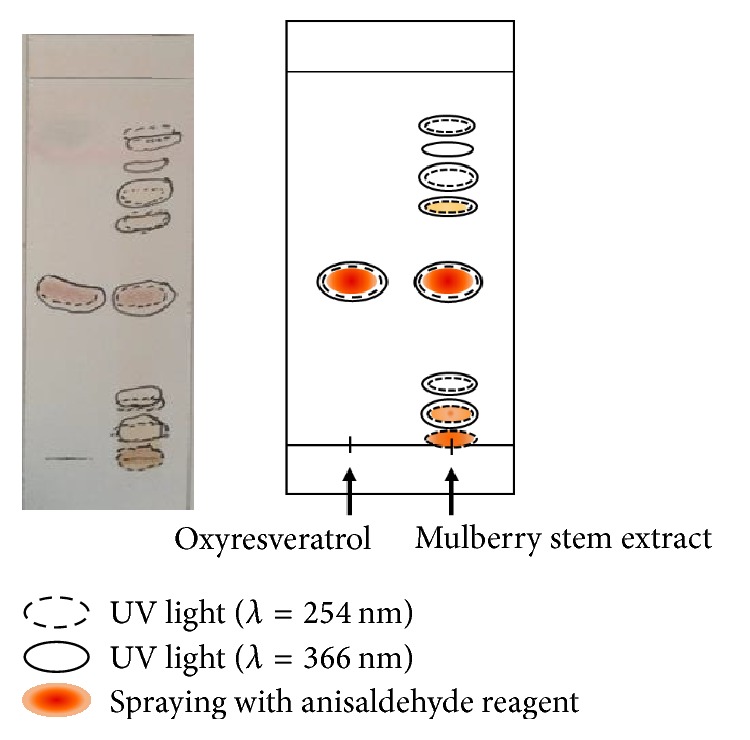
TLC fingerprint of mulberry stem extract. Standard oxyresveratrol was used as a marker. Mobile phase system was methanol : chloroform (15 : 85% v/v).

TLC analysis was performed to conduct quality control of natural herbal product containing complex mixtures of compounds like in the case of mulberry stem extract. Oxyresveratrol, one of the bioactive compounds found in mulberry stem extract, was used as a marker. Observing under UV light at 254 nm and 366 nm, the results showed that mulberry stem extract showed eight spots with *R*_*f*_ values 0.02, 0.09, 0.16, 0.44, 0.64, 0.71, 0.78, and 0.87, while oxyresveratrol showed only clear one spot with *R*_*f*_ value 0.44 by comparing *R*_*f*_ value. It was clearly shown that a reddish spot observed in mulberry stem extract after spraying TLC plate with anisaldehyde reagent with *R*_*f*_ value of 0.44 was oxyresveratrol (Figure 1).

### 3.3. Cell Viability

**Figure 2:**
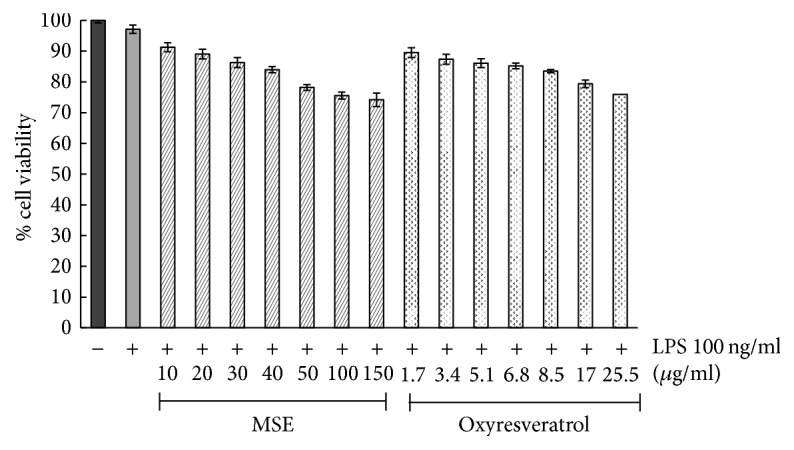
The percentage viability of RAW 264.7 cells which was untreated (negative control), treated with only 100 ng/ml LPS (positive control), and treated with various concentrations of MSE and oxyresveratrol in the presence of 100 ng/ml LPS for 24 h. The viability of cells was determined by MTT assay. The data were represented as means ± SD of three independent experiments.

To ensure that the cells were healthy before performing the bioactivity assays and the tested concentrations were not toxic to the cells, cell viability after treatment with various concentrations of the MSE was determined. The concentrations of the MSE that yielded cell viability higher than 80% were the main aim of this experiment. The RAW 264.7 cell viability slightly decreased with increasing the concentration of the MSE from 10 to 150 *µ*g/ml (Figure 2). Identifying the concentrations of the MSE that yielded cell viability higher than 80%, it was found to be in the range of 10 to 40 *µ*g/ml and the concentrations of the MSE selected for subsequent studies were 20 and 40 *µ*g/ml. Oxyresveratrol was included in this study as the marker of the MSE. An equivalent amount of oxyresveratrol in each concentration of the extract tested (for example, equivalent amount of oxyresveratrol in the extract 100 *µ*g/ml was 17 *µ*g/ml) was also evaluated for the effect on cell viability. The results were shown to be similar to those observed in the MSE-treated cells (Figure 2). The anti-inflammatory effects of oxyresveratrol were also confirmed in subsequent studies, in which the selected concentrations of oxyresveratrol were 5 and 10 *µ*g/ml.

### 3.4. Anti-Inflammatory Activity of the MSE

*M. alba* extract has been demonstrated to exhibit anti-inflammatory activity, through the inhibition of iNOS, NO, and COX [10–15]. However, most of the extract tested was prepared from leaves [10, 11, 14] and root bark [12, 13, 15]. In our study, anti-inflammation activity of the extract prepared from mulberry stems was investigated.

In an LPS-stimulated mouse macrophage RAW 264.7 cells model, the NF-*κ*B signaling pathway plays a crucial role in regulating inflammation through the transcription of iNOS, COX, and cytokine genes. Found in the cytoplasm of resting cells, dimer NF-*κ*B is normally confined to an inactive cytoplasmic complex through binding to an inhibitory protein, I-*κ*B, which masks its nuclear localization signal. Exposure of cells to external proinflammatory stimuli such as mitogens, inflammatory cytokines, and LPS causes rapid I-*κ*B phosphorylation by I-*κ*B kinase (IKK), resulting in free and subsequent nuclear translocation of NF-*κ*B [16, 17]. In the nucleus, NF-*κ*B induces the transcription of a large variety of target proinflammatory genes including iNOS and COX-2. iNOS then further facilitates the conversion of L-arginine to L-citrulline and a large amount of NO, while COX-2 catalyzes the conversion of arachidonic acid to prostaglandins. Prostaglandins (PGs), especially PGE_2_ and PGI_2_, cause vasodilation that contribute to the vascular signs of inflammation and potentiate edema formation [18, 19].

#### 3.4.1. Effect of the MSE on the Expression of iNOS mRNA and Protein

**Figure 3:**
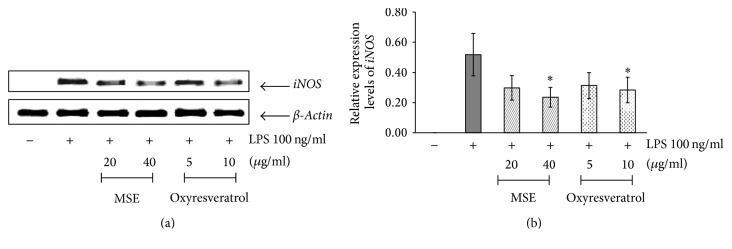
The effects of MSE and oxyresveratrol on the expression of iNOS mRNA in RAW 264.7 cells. The untreated cells were used as a negative control and the cells treated with only 100 ng/ml LPS were served as a positive control. (a) RT-PCR analysis for detection of iNOS mRNA expression in LPS-stimulated RAW 264.7 cells which were pretreated with MSE at the concentrations of 20 and 40 *µ*g/ml or oxyresveratrol at the concentrations 5 and 10 *µ*g/ml for 2 h. (b) The relative expression of iNOS mRNA compared to *β*-actin. The data were represented as means ± SD of three independent experiments. The means marked with *∗* are significantly different (*P* < 0.05) from that of the cells treated with only LPS.

**Figure 4:**
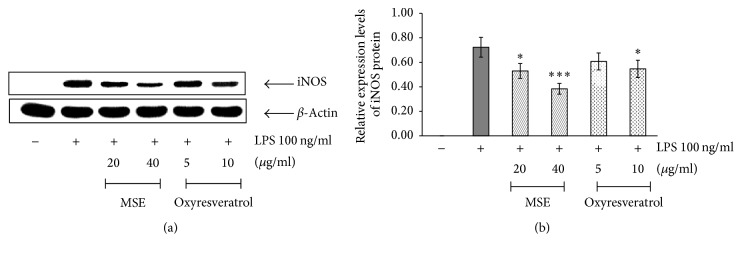
The effects of MSE and oxyresveratrol on the expression of iNOS protein in RAW 264.7 cells. The untreated cells were used as a negative control and the cells treated with only 100 ng/ml LPS served as a positive control. (a) Immunoblotting analysis for determination the expression of iNOS protein in LPS-stimulated RAW 264.7 cells which were pretreated with MSE at the concentrations of 20 and 40 *µ*g/ml or oxyresveratrol at the concentrations 5 and 10 *µ*g/ml for 2 h. (b) The relative expression of iNOS protein compared to *β*-actin. The data were represented as means ± SD of three independent experiments. The means marked with *∗*, *∗∗∗* are significantly different (*P* < 0.05) and (*P* < 0.001), respectively, from that of the cells treated with only LPS.

The anti-inflammatory effects of the MSE at the concentrations of 20 and 40 *μ*g/ml were investigated through the inhibition on the expression of iNOS mRNA and its protein as well as the production of NO. Oxyresveratrol (marker) at the concentrations of 5 and 10 *μ*g/ml was also tested. *β*-Actin was used as an internal standard for RT-PCR assay. As expected, the expression of iNOS mRNA and its protein was detected by stimulating RAW 264.7 cells with LPS (Figures 3 and 4). The expression level of iNOS in cells treated with the MSE at the concentration of 40 *μ*g/ml was decreased by 50% compared with that in cells treated with LPS alone (Figure 3). Similar results were observed in cells treated with oxyresveratrol.

Western blotting was further performed to confirm the effects of the MSE on iNOS protein expression. The results showed that iNOS protein was significantly decreased in untreated RAW 264.7 cells comparing with LPS-treated RAW 264.7 cells (Figure 4). Therefore, the validity of the iNOS protein assay was again confirmed. The MSE at the concentrations of 20 (*P* < 0.05) and 40 (*P* < 0.001) *µ*g/ml and oxyresveratrol at 10 (*P* < 0.05) *µ*g/ml significantly inhibited the expression of iNOS protein compared with the cells treated only with LPS.

#### 3.4.2. Effect of the MSE on NO Production

The inducible forms of NOS are the most important proinflammatory enzymes responsible for increasing the levels of NO. Therefore, the effect of tested compounds on the inhibition of NO production was further investigated. However, NO in the biological matrix is very unstable and rapidly oxidizes to nitrite (NO_2_^−^) and thus the measurement of nitrite is routinely used as an index of NO production.

**Figure 5:**
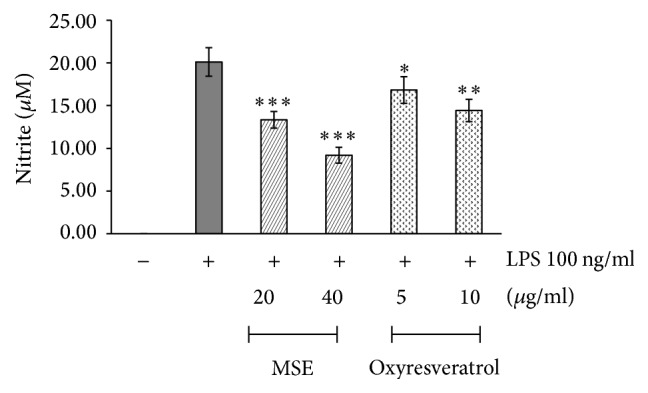
The effects of MSE and oxyresveratrol on the production of nitrite in LPS-stimulated RAW 264.7 cells which were untreated (negative control), treated with only 100 ng/ml LPS (positive control), pretreated with MSE at the concentrations of 20 and 40 *µ*g/ml or oxyresveratrol at the concentrations of 5 and 10 *µ*g/ml for 2 h, and then treated with 100 ng/ml LPS for 16 h. The supernatant was collected and determined by Griess reaction assay. The data were represented as means ± SD of three independent experiments. The means marked with *∗*, *∗∗*, *∗∗∗* are significantly different (*P* < 0.05), (*P* < 0.005), and (*P* < 0.001), respectively, from that of the cells treated with only LPS.

For the untreated RAW 264.7 cells, the concentration of nitrite could not be detected (Figure 5). Once the cells were stimulated with LPS, a high concentration of nitrite was produced. The MSE at concentrations of 20 and 40 *μ*g/ml were shown to inhibit nitrite production by 34% and 54%, respectively, and oxyresveratrol at concentrations of 5 and 10 *μ*g/ml was able to inhibit nitrite production by 16% and 28%, respectively (Figure 5).

#### 3.4.3. Effect of the MSE on COX-2 mRNA Expression

**Figure 6:**
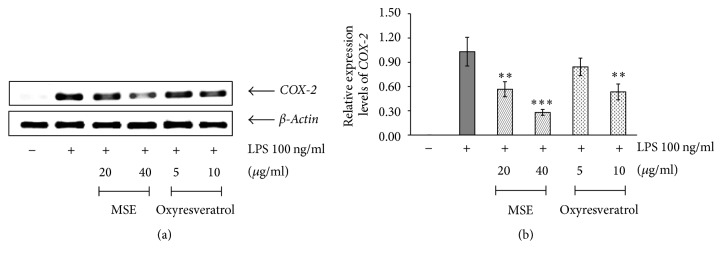
The effects of MSE and oxyresveratrol on the expression of COX-2 mRNA in RAW 264.7 cells. The untreated cells were used as a negative control and the cells treated with only 100 ng/ml LPS served as a positive control. (a) RT-PCR analysis for detection of COX-2 mRNA expression in LPS-stimulated RAW 264.7 cells which were pretreated with MSE at the concentrations of 20 and 40 *µ*g/ml or oxyresveratrol at the concentrations of 5 and 10 *µ*g/ml for 6 h. (b) The relative expression of COX-2 mRNA compared to *β*-actin. The data were represented as means ± SD of three independent experiments. The means marked with *∗∗*, *∗∗∗* are significantly different (*P* < 0.005) and (*P* < 0.001), respectively, from that of the cells treated with only LPS.

As shown in Figure 6, the MSE was able to inhibit the expression of COX-2 mRNA in LPS-activated RAW 264.7 cells. The inhibitory activity of the MSE at 40 *μ*g/ml was shown to be higher than that of the MSE at 20 *μ*g/ml. The expression level of COX-2 mRNA in cells treated with the MSE at 40 *μ*g/ml decreased by 70%, compared with that in cells treated with LPS alone. Similar results were observed when LPS-activated RAW 264.7 cells were incubated with oxyresveratrol.

The results observed in this study clearly showed that the MSE could inhibit NO production, through the inhibition of both iNOS mRNA and protein expression. In addition, the extract was shown to suppress the expression of COX-2 mRNA in LPS-stimulated RAW 264.7 cells. The inhibition effect on production of iNOS, COX-2, and NO of the MSE at 40 *µ*g/ml was found to be higher than that of the MSE at 20 *µ*g/ml. The MSE showed more potent inhibitory effects than oxyresveratrol at an equivalent amount of oxyresveratrol (the MSE at 40 *µ*g/ml equivalent to 6.8 *µ*g/ml). It has been recently reported that the stems of* M. alba* contained flavonoids (morusin), stilbenoids (mulberroside, resveratrol, and oxyresveratrol), and coumarins [1]. These polyphenols were shown to possess anti-inflammatory effects [20–22]. We also determined the amounts of total phenolic of the extract and it was 34.87 ± 1.12 *μ*mol GAE/mg extract. Therefore, these polyphenols, other than oxyresveratrol, may explain the superior anti-inflammatory effect of the MSE. Although the precise molecular mechanisms responsible for the anti-inflammatory activities of the MSE cannot be explained in the present study, oxyresveratrol which is an active component presented in the extract has been reported to exhibit its anti-inflammatory effect via the inhibition of the NF-*κ*B and the MAPK pathways [2, 3]. However, further elucidation of the insight mechanisms involved the anti-inflammatory effect of this plant extract is required.

## 4. Conclusions

This study is the first to demonstrate the potential anti-inflammatory effects of the extract prepared from mulberry stems on LPS-induced RAW 264.7 cells. The investigation found that concentrations at 20 and 40 *µ*g/ml had no toxic effect. The results suggest that the stem extract mediates its anti-inflammatory activities through the inhibition of iNOS, NO, and COX-2 production. Further studies are necessary to fully define the precise mechanisms involved.

## Figures and Tables

**Table 1:** The primer sequences used for PCR amplification.

Gene	Primer sequence (5′-3′)	Product size (bp)	PCR condition	Reference
*iNOS*	GCA GAA TGT GAC CAT CAT GG ACA ACC TTG GTG TTG AAG GC	414	$\begin{array}{cc} \begin{array}{c} 94^{{}^{\circ}}C,\;\;5\;min\;\;\;\;\\ \left.\begin{array}{c} 94^{{}^{\circ}}C,\;\;45\;sec\\ 60^{{}^{\circ}}C,\;\;1\;min\\ 72^{{}^{\circ}}C,\;\;1\;min \end{array}\right] \end{array} & 30\;\;cycles \end{array}$	[8]

*COX-2*	AGA AGG AAA TGG CTG CAG AA GCT CGG CTT CCA GTA TTG AG	194	$\begin{array}{cc} \begin{array}{c} 94^{{}^{\circ}}C,\;\;5\;min\;\;\;\;\\ \left.\begin{array}{c} 94^{{}^{\circ}}C,\;\;30\;sec\\ 54^{{}^{\circ}}C,\;\;30\;sec\\ 72^{{}^{\circ}}C,\;\;30\;sec \end{array}\right] \end{array} & 30\;\;cycles \end{array}$	This study

*β-Actin*	CCA GAG CAA GAG AGG TAT CC CTG TGG TGG TGA AGC TGT AG	436	$\begin{array}{cc} \begin{array}{c} 94^{{}^{\circ}}C,\;\;5\;min\;\;\;\;\\ \left.\begin{array}{c} 94^{{}^{\circ}}C,\;\;45\;sec\\ 58^{{}^{\circ}}C,\;\;1\;min\\ 72^{{}^{\circ}}C,\;\;1\;min \end{array}\right] \end{array} & 30\;\;cycles \end{array}$	[8]

## References

[1] Chan E. W.-C., Lye P.-Y., Wong S.-K. (2016). Phytochemistry, pharmacology, and clinical trials of Morus alba. *Chinese Journal of Natural Medicines*.

[2] Chung K.-O., Kim B.-Y., Lee M.-H. (2003). *In-vitro* and *in-vivo* anti-inflammatory effect of oxyresveratrol from *Morus alba* L. *Journal of Pharmacy and Pharmacology*.

[3] Lee H. S., Kim D. H., Hong J. E., Lee J.-Y., Kim E. J. (2015). Oxyresveratrol suppresses lipopolysaccharide-induced inflammatory responses in murine macrophages. *Human and Experimental Toxicology*.

[4] Thongsuk P. (2007). *In vitro and clinical study of mulberry extract for skin whitening product [M.S. thesis]*.

[5] Laveti D., Kumar M., Hemalatha R. (2013). Anti-inflammatory treatments for chronic diseases: a review. *Inflammation & Allergy—Drug Targets*.

[6] Chen Y.-C., Tien Y.-J., Chen C.-H. (2013). *Morus alba* and active compound oxyresveratrol exert anti-inflammatory activity via inhibition of leukocyte migration involving MEK/ERK signaling. *BMC Complementary and Alternative Medicine*.

[7] Yhirayha C. (2013). *Formulation and skin penetration study of lyotropic liquid crystal incorporating mulberry stem (Morus alba L.) extract [M.S. thesis]*.

[8] Pudla M., Limposuwan K., Utaisincharoen P. (2011). Burkholderia pseudomallei-Induced expression of a negative regulator, sterile-*α* and Armadillo motif-containing protein, in mouse macrophages: a possible mechanism for suppression of the MyD88-independent pathway. *Infection and Immunity*.

[9] Soonthornsit N., Pitaksuteepong T. Microemulsion formulation containing mulberry stem extract.

[10] Hong C. H., Hur S. K., Oh O.-J., Kim S. S., Nam K. A., Lee S. K. (2002). Evaluation of natural products on inhibition of inducible cyclooxygenase (COX-2) and nitric oxide synthase (iNOS) in cultured mouse macrophage cells. *Journal of Ethnopharmacology*.

[11] Choi E.-M., Hwang J.-K. (2005). Effects of *Morus alba* leaf extract on the production of nitric oxide, prostaglandin E_2_ and cytokines in RAW264.7 macrophages. *Fitoterapia*.

[12] Yang Z.-G., Matsuzaki K., Takamatsu S., Kitanaka S. (2011). Inhibitory effects of constituents from Morus alba var. multicaulis on Differentiation of 3T3-L1 cells and nitric Oxide Production in RAW264.7 Cells. *Molecules*.

[13] Lim H. J., Jin H.-G., Woo E.-R., Lee S. K., Kim H. P. (2013). The root barks of *Morus alba* and the flavonoid constituents inhibit airway inflammation. *Journal of Ethnopharmacology*.

[14] Park E., Lee S.-M., Lee J. E., Kim J.-H. (2013). Anti-inflammatory activity of mulberry leaf extract through inhibition of NF-*κ*B. *Journal of Functional Foods*.

[15] Zelova H., Hanakova Z., Cermákova Z. (2014). Evaluation of anti-inflammatory activity of prenylated substances isolated from *Morus alba* and *Morus nigra*. *Journal of Natural Products*.

[16] Abate A., Oberle S., Schröder H. (1998). Lipopolysaccharide-induced expression of cyclooxygenase-2 in mouse macrophages is inhibited by chloromethylketones and a direct inhibitor of NF-*κ*B translocation. *Prostaglandins and Other Lipid Mediators*.

[17] Lee S.-H., Kwak C.-H., Lee S.-K. (2016). Anti-inflammatory effect of ascochlorin in lps-stimulated raw 264.7 macrophage cells is accompanied with the down-regulation of iNOS, COX-2 and proinflammatory cytokines through NF-*κ*B, ERK1/2, and p38 signaling pathway. *Journal of Cellular Biochemistry*.

[18] Parente L. (2001). Pros and Cons of Selective Inhibition of COX-2 versus Dual Lipoxygenase/COX Inhibition: Is Two Better than One?. *The Journal of Rheumatology*.

[19] Das U. N., Undurti N. D. (2010). Cyclooxygenase (COX), lipoxygenase (LO) pathways and generation of lipoxins, resolvins, protections and maresins. *Molecular Basis of Health and Disease*.

[20] Bellik Y., Boukraa L., Alzahrani H. A. (2012). Molecular mechanism underlying anti-inflammatory and anti-allergic activities of phytochemicals: an update. *Molecules*.

[21] Rivière C., Krisa S., Péchamat L. (2014). Polyphenols from the stems of *Morus alba* and their inhibitory activity against nitric oxide production by lipopolysaccharide-activated microglia. *Fitoterapia*.

[22] Das S., Das D. K. (2007). Anti-inflammatory responses of resveratrol. *Inflammation and Allergy-Drug Targets*.

